# HRTFs Measurement Based on Periodic Sequences Robust towards Nonlinearities in Automotive Audio †

**DOI:** 10.3390/s23031692

**Published:** 2023-02-03

**Authors:** Stefania Cecchi, Valeria Bruschi, Stefano Nobili, Alessandro Terenzi, Alberto Carini

**Affiliations:** 1 Department of Information Engineering, Università Politecnica delle Marche, 60131 Ancona, Italy; 2 Department of Engineering and Architecture, University of Trieste, 34127 Trieste, Italy

**Keywords:** head related transfer functions, HRTFs measurement, perfect periodic sequences, orthogonal periodic sequences, automotive audio

## Abstract

The head related transfer functions (HRTFs) represent the acoustic path transfer functions between sound sources in 3D space and the listener’s ear. They are used to create immersive audio scenarios or to subjectively evaluate sound systems according to a human-centric point of view. Cars are nowadays the most popular audio listening environment and the use of HRTFs in automotive audio has recently attracted the attention of researchers. In this context, the paper proposes a measurement method for HRTFs based on perfect or orthogonal periodic sequences. The proposed measurement method ensures robustness towards the nonlinearities that may affect the measurement system. The experimental results considering both an emulated scenario and real measurements in a controlled environment illustrate the effectiveness of the approach and compare the proposed method with other popular approaches.

## 1. Introduction

Head related transfer functions (HRTFs) are mathematical functions that model the acoustic path between a sound source and the ears of the listener. They can be measured using artificial head simulators or listeners equipped with a pair of in-ear microphones. The HRTFs take into account the human ear’s properties and the sound localization cues, i.e., the interaural level difference (ILD), the interaural time difference (ITD), and the spectral attributes needed by the human brain to localize a sound source in the space. This paper is focused on making the HRTF measurement method robust towards the nonlinearities proposed in [1] and presents new extended experimental results.

The HRTFs are employed in many audio applications [2]. In particular, they are essential in immersive audio rendering, where the audio virtualization is achieved by the real-time convolution of the input signal with the selected HRTFs. They are also used in crosstalk cancellation systems to eliminate the crosstalk signals generated by the loudspeakers’ superposition during the binaural reproduction.

Nowadays, the car is one of the most used audio listening environments, so the application of immersive systems also becomes appealing for the automotive scenario. In [3], the HRTFs are used inside the car to obtain an immersive reproduction exploiting binaural techniques, while in [4], the HRTFs are needed to recreate a virtual environment in order to evaluate the human being’s perception of a sound system. In recent years, studies on spatial sound and auralization systems in the car cockpit have been investigated and implemented, as reported in [5,6].

Focusing on the HRTFs measurement methodology, several approaches can be found in the literature [2]. The classical methods are based on the deconvolution approach and can be categorized depending on the excitation signal. The most used signals are the pseudo random sequences [7,8,9,10,11,12,13,14,15,16] and the sweep signals [17,18,19,20,21]. The pseudo-random sequence is a deterministic discrete time signal and it includes the maximum length sequence (MLS) [9,10,11,12,13], the inverse repeated sequence (IRS) [14,15] and the Golay codes [16]. Otherwise, a sweep signal is a continuous signal whose frequency changes with time and it may be a linear sweep [20] or an exponential sweep [21]. A detailed comparison of different impulse response measurement approaches discussed so far can be found in [22]. It should be noted that all these approaches suffer from any nonlinearity present in the measurement system, such as those affecting the power amplifier or the loudspeaker. The nonlinearities can generate evident artifacts, such as the spikes observed in the MLSs measurement [23], or sometimes less evident alterations of the measured impulse response, as in the exponential sweeps [24,25,26].

An alternative to deconvolution techniques is the adaptive filtering approach, firstly applied to HRTFs measurement by Enzner in [27] and then also used in other more recent research [28,29]. This method is mostly based on the normalized least mean square (NLMS) algorithm, characterized by high performance and simplicity of implementation [30].This method can also be affected by nonlinearities in the measurement system [27,28,29]. The HRTF measurement can be affected by different problems. Together with the already-mentioned nonlinear distortions introduced by the electro-acoustic system, the measurement is influenced by the characteristics of the sound sources, especially in near-field measurements [2], and environmental disorders, such as external noise, sound reflections, and temperature changes [31]. The use of a controlled environment, such as an anechoic chamber, can avoid some of these problems, e.g., noise, reflections, and temperature changes, while the nonlinear distortions can be prevented by choosing an effective measurement method and stimuli [32,33,34,35,36,37,38,39].

The authors have recently proposed different methods for the robust measurement of the room impulse response in the presence of nonlinearities in the electro-acoustic system. In these methods, the measurement system is directly modeled as a nonlinear filter and its linear part is estimated. A first approach was proposed in [33,35,36,38], where a Legendre nonlinear (LN) filter [33,35] or a Wiener nonlinear (WN) filter [36,38] is used to model the measurement system. LN and WN filters are orthogonal polynomial filters that admit PPSs, i.e., periodic sequences that guarantee the perfect orthogonality of the basis functions over a period. Using PPSs for LN or WN filters, the linear part of the system, i.e., the first-order kernel, can be measured with the cross-correlation method, i.e., computing the cross-correlation of the output with the PPS. At the same time, the influence of any nonlinear term can be rejected. Later, Ref. [40] introduced the OPSs, which were applied to the robust room impulse response measurement in [37,39]. The OPSs allow the identification of a broad class of nonlinear filters, the functional link polynomial (FLiP) filters. The FLiP filter class includes LN and WN filters and also the well-known Volterra filters. Given a persistently exciting periodic input sequence, an OPS is a periodic sequence that, cross-correlated with the system output, provides one of the so-called “diagonals” of the FLiP filter [40]. In [39], the OPS has been used to measure the room impulse response from the first-order kernel of a Volterra filter. OPSs can be more easily developed than PPSs but they are more sensitive to noise than PPSs. In [1], PPSs and OPSs were applied for the first time to the HRTFs measurement, considering a car environment.

Table 1 provides a summary of the HRTFs measurement methods proposed in the literature divided by category and indicates their robustness towards nonlinearities.

Starting from the results of [1], this paper presents an extension of the experimental tests, obtained by adding new measurements and applying different types of nonlinearities. The obtained results are compared with other state-of-the-art HRTFs measurement techniques to prove the effectiveness of the proposed approach.

The paper is organized as follows. Section 2 describes the proposed methodology and introduces in more detail the functional link polynomial (FLiP) filters, the periodic perfect sequences (PPSs), and the orthogonal perfect sequences (OPSs). Section 3 shows the experimental results, obtained through experiments carried out in a real scenario (Section 3.1) and in an emulated nonlinear scenario (Section 3.2), comparing the proposed measurement methods with the state-of-the-art. Finally, conclusions are reported in Section 4.

## 2. FLiP Filters, PPSs, and OPSs

In the proposed methodology, the measurement system is modelled as a nonlinear system, which could be a Volterra, a Legendre, or a Wiener nonlinear filter. For generality, we assume the nonlinear system belongs to the class of FLiP nonlinear filters, which comprises all the previously mentioned filters and many others. Our objective is the measurement of the first-order kernel of the nonlinear filter, which corresponds to the filter linear part. The measurement will be performed using PPSs or OPSs. Measuring the HRTFs with these sequences allows us to capture the acoustic transfer function, apart from the effect of the loudspeaker and microphone, which is neglected as usual. In this section, we first review the FLiP filters, and then we describe how PPSs and OPSs can be built and how they can be used for impulse response estimation. A comparison between PPSs and OPSs will also follow.

### 2.1. FLiP Filters

FLiP filters [41] are a broad class of nonlinear filters that can arbitrarily well approximate any discrete-time, time-invariant, finite memory, continuous nonlinear system,

(1) y(n)=f[x(n),x(n−1),…,x(n−N+1)],

where *f* is a continuous *N*-dimensional function, *N* is the system memory length, and the input signal x(n) is defined in the compact [−1,+1].

FLiP filters are linear-in-the-parameter nonlinear filters, i.e., are a linear combination of basis functions. They are derived by considering a set of one-dimensional basis functions, the “generating” polynomials, which are assumed to satisfy the Stone–Weierstrass theorem [42]:

(2) $$\{g_{0}\left(\xi \right),g_{1}\left(\xi \right),g_{2}\left(\xi \right),g_{3}\left(\xi \right),\ldots \},$$

where $g_{0}\left(\xi \right)$ is a basis function of order 0, usually the constant 1, $g_{1}\left(\xi \right)$ is a basis function of order 1 and very often is the linear mononial ξ, and in general $g_{i}\left(\xi \right)$ for all i∈N is a basis function of order *i*, with all even basis functions $g_{2i}\left(\xi \right)$ that are even and all odd basis functions $g_{2i+1}\left(\xi \right)$ that are odd. The FLiP filter basis functions are formed by first writing the one-dimensional basis functions for ξ=x(n),x(n−1),…,x(n−N+1) and then multiplying the functions $g_{i}$ of different variables by each other in any possible manner, taking care of avoiding repetitions, as in the triangular representation of Volterra filters. The order of a FLiP basis function is the sum of the orders of the constituent factors $g_{i}$. The diagonal number of a basis function is defined as the maximum time difference between the input samples involved in the factors. For example, the basis function $g_{3}\left[x\left(n\right)\right]g_{1}\left[x\left(n-3\right)\right]$ has order 4 and diagonal number 3.

A FLiP filter of order *K*, diagonal number *D*, and memory *N* is given by the linear combination of all basis functions obtained with this procedure until order *K* and maximum diagonal number *D*. For example, a FLiP filter of order 2, diagonal number *D*, memory *N* has the following input–output relationship:

(3) $$\begin{array}{ccc} y(n) & \;=\; & h_{0}+\sum_{m=0}^{N-1}h_{1,m}g_{1}\left[x\left(n-m\right)\right]\\ & & +\sum_{m=0}^{N-1}h_{2,0,m}g_{2}\left[x\left(n-m\right)\right]\\ & & +\sum_{i=1}^{D}\sum_{m=0}^{N-1-D}h_{2,i,m}g_{1}\left[x\left(n-m\right)\right]g_{1}\left[x\left(n-m-i\right)\right], \end{array}$$

and in general a FLiP filter of order *K*, diagonal number *D*, and memory *N*, has the following input–output relationship:

(4) $$\begin{array}{ccc} y(n) & = & h_{0}+\sum_{m=0}^{N-1}h_{1,m}f_{1}\left[x\left(n-m\right)\right]+\\ & & \sum_{r=1}^{K}\sum_{i_{1}=0}^{D}\sum_{i_{2}=i_{1}}^{D}\ldots \sum_{i_{r-1}=i_{r-2}}^{D}\sum_{m=0}^{N-i_{r-1}}h_{r,i_{1},i_{2},\ldots ,i_{r-1}}\cdot \\ & & f_{r,i_{1},i_{2},\ldots ,i_{r-1}}[x\left(n-m\right),x\left(n-m-i_{1}\right),\\ & & \;x\left(n-m-i_{2}\right)...,x\left(n-m-i_{r-1}\right)]. \end{array}$$

In (4), $h_{0}$ is a constant term and is usually neglected in audio applications, $h_{1,m}$ is the first order kernel, $f_{1}\left[\cdot \right]=g_{1}\left[\cdot \right]$ is the first order basis function, $h_{r,i_{1},\ldots ,i_{r-1}}$ is the *r*-th order kernel, and $f_{r,i_{1},\ldots ,i_{r-1}}\left[\cdot \right]$ are the basis functions of order *r*, i.e., polynomials of order *r* that are product of the “generating" polynomials $g_{i}$ in the arguments. Every choice of the generating polynomials $g_{i}$ takes to a different family of FLiP filters. In Volterra filters, the generating polynomials are the monomials $g_{i}=x^{i}$. In LN filters, they are Legendre polynomials, and in WN filters, they are Hermite polynomials. In all these filters, $g_{1}\left(\xi \right)=\xi$ and the filter in (4) is composed of a linear filter,

$$\sum_{m=0}^{N-1}h_{1,m}x\left(n-m\right),$$

plus a combination of higher order polynomial basis functions.

LN and WN filters are orthogonal FLiP filters. The LN basis functions are orthogonal for a white uniform distribution of the input samples, while the WN basis functions are orthogonal for a Gaussian distribution of the input samples. Thanks to their orthogonality properties, LN and WN filters admit PPSs, i.e., periodic sequences that guarantee the same orthogonality of the basis functions on a finite period [43]. In contrast, the basis functions of Volterra filters are non orthogonal for any input sample distribution and thus they do not admit PPSs. Nevertheless, they admit OPSs as detailed in the following.

### 2.2. Perfect Periodic Sequences

PPSs for LN and WN filters can be developed as described in [43,44] by considering a set of variables representing the samples of the periodic sequence and imposing the orthogonality of all basis functions of the filter, i.e., imposing that for any two different basis functions in (4), shortly denoted as $f_{i}\left(n\right)$ and $f_{j}\left(n\right)$, the cross-correlation is zero, i.e.,

(5) $$<f_{i}\left(n\right)f_{j}\left(n\right){>}_{{}_{P}}=0,$$

where $<\cdot {>}_{{}_{P}}$ indicates the sum of the terms between angular brackets for *n* ranging over a period *P* of the sequence. Imposing (5) for all possible couples of basis functions in (4), a system of nonlinear equations is obtained and for a sufficiently large period *P* the system is underdetermined. A solution for this nonlinear system has always been found using the Newton–Raphson method.

Using a PPS input signal, all basis functions are mutually orthogonal. Since x(n−m) for each *m* in [0,N−1] is a basis function, it becomes possible to measure the linear kernel $h_{1,m}$ with the cross-correlation method, i.e., computing the cross-correlation between the system output and the PPS input sequence:

(6) $$h_{1,m}=\frac{<y\left(n\right)x\left(n-m\right){>}_{{}_{P}}}{<x^{2}\left(n\right){>}_{{}_{P}}},$$

as can be easily proved by replacing y(n) with (4).

### 2.3. Orthogonal Periodic Sequences

Many families of FLiP filters, including the Volterra filters, are non-orthogonal and thus do not admit PPSs. Nevertheless, they can still be identified with the cross-correlation method using OPSs. Given any persistently exiting periodic input sequence x(n) of sufficiently large period *P*, an orthogonal periodic sequence z(n) of period *P* can be developed such that

(7) $$h_{1,m}=<y\left(n\right)z\left(n-m\right){>}_{{}_{P}}.$$

In [40], the OPS has been developed imposing

(8) $$<x\left(n\right)z\left(n\right){>}_{{}_{P}}=1,$$

and at the same time the orthogonality of z(n) with all other basis functions $f_{i}\left(n\right)\neq x\left(n\right)$,

(9) $$<x\left(n\right)f_{i}\left(n\right){>}_{{}_{P}}=0.$$

In this way, a linear equation system in the variables z(n), for *n* ranging over a period *P*, is obtained and for sufficiently large *P*, the system is underdetermined and always admits a solution.

### 2.4. A Comparison between PPSs and OPSs

For the same memory length *N*, order *K*, diagonal number *D*, and period *P*, computing an OPS is much simpler and faster than a PPS. The OPS requires solving a linear equation system, while the PPS requires solving a nonlinear system. In OPSs, the input signal is chosen a priori and can be any persistently exciting sequence, even a quantized sequence with a reduced number of levels. For example, the input sequence can be formed by samples having a Gaussian or uniform distribution. The same input sequence can be used to develop OPSs for Volterra, LN, and WN filters. In PPSs, in the estimation of $h_{1,m}$ the input sequence and the orthogonal sequence coincide and are the PPS itself. A sufficiently large number of levels must be used in the sequence quantization otherwise the orthogonality between the basis functions is lost. Moreover, OPSs and PPSs have different behavior in the presence of noise. To compare sequences of different periods on equal terms, the noise gain has been introduced in [40] and is defined as

(10) $$G_{\nu }=\frac{MSD}{E[\nu {\left(n\right)}^{2}]}<x^{2}\left(n\right){>}_{{}_{P}},$$

where MSD is the mean square deviation in the coefficient estimate, i.e., $MSD=E[{\left({\tilde{h}}_{1,m}-h_{1,m}\right)}^{2}]$, with ${\tilde{h}}_{1,m}$ the true value and $h_{1,m}$ the estimated one, and $E[\nu {\left(n\right)}^{2}]$ is the noise variance. It can be proved that PPSs always have $G_{\nu }=1$. On the contrary, for OPSs when the period *P* is small, i.e., close to the minimum value allowed by the conditions of the linear equation system, the noise gain $G_{\nu }$ assumes very large values that make the identification with OPSs useless. Nevertheless, for large periods, $G_{\nu }$ assumes reasonable values that make the identification with OPSs feasible and useful. We have found experimentally that, for the same values of *N*, *K* and *D*, the period of the OPS should be twice that of a PPS to obtain reasonable values of $G_{\nu }$ in the estimation of the first order kernel.

Eventually, we must point out that PPSs can be applied to the identification of the first order kernel only of orthogonal FLiP filters, e.g., LN and WN filters, while the first order kernel of Volterra filters can be estimated with the cross-correlation method only using OPSs. Moreover, the first order kernel of the Volterra model coincides with the impulse response of the system when the input signal amplitude tends to zeros, which is not the case for LN and WN filters.

### 2.5. Computational Cost of the Identification with PPSs and OPSs

The computational cost of identification with PPSs and OPSs using equations (6) and (7), respectively, is of order NP operations. In reality, the formulas can be implemented in the FFT domain with a computational cost of $P\;\log_{2}P$ operations. Thus, these techniques have the same computational cost as the methods based on MLSs or exponential sweeps but introduce robustness toward nonlinearities since they estimate the linear component of a polynomial filter (a Volterra, LN, or WN filter). More properly, the computational complexity of the proposed techniques should be compared with that of the least-squares (LS) technique usually used to identify the coefficients of polynomial filters. The LS technique has a computational cost of $M^{2}L$ operations, with *M* the number of nonlinear coefficients, which in general is much larger than the impulse response length *N*, M≫N. Thus, the proposed techniques provide a great computational complexity saving with respect to the LS technique, and also a large advantage in memory usage.

## 3. Experimental Results

Two types of experiments have been performed. The first one was based on the validation of the proposed approach in a real scenario, i.e., a car equipped with a binaural mannequin. The second one was based on an emulated scenario in order to create different controlled distortion levels through specific devices since the considered car environment has shown a low level of nonlinearity.

### 3.1. Real Scenario

**Figure 1:**
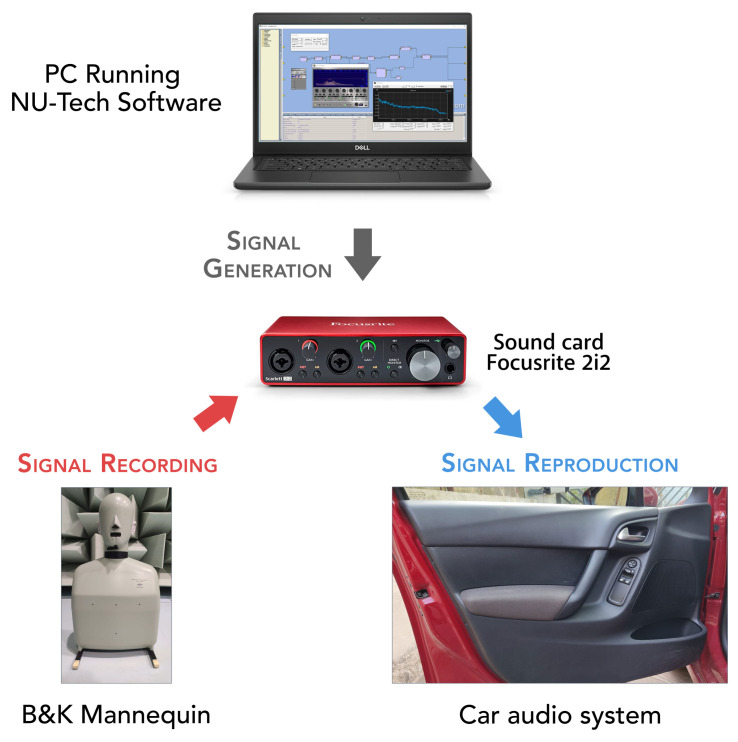
Overall scheme of the acquisition procedure for the real scenario.

**Figure 2:**
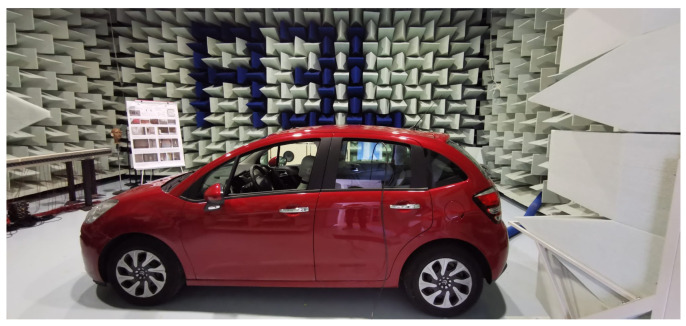
Car used for the experiments with the B&K mannequin. The experiments were performed inside the semi-anechoic chamber of the A3Lab group at Università Politecnica delle Marche.

For the real scenario, the HRTFs measurement was performed according to the scheme reported in Figure 1. In particular, for the recording, a Bruel&Kjaer Head and Torso Simulator Type 4128, with right and left Ear Simulators (Type 4158 and 4159) connected to a Sound Card Focusrite 2i2 through the Bruel&Kjaer microphone preamplifier Type 2829, was used. For the reproduction, the car sound system was used, exploiting the auxiliary car audio port for the connection with the same sound card used for the recordings. The NU-Tech software [45] exploiting ASIO drivers was used on a PC for reproduction and acquisition synchronization. Finally, to ensure a low environmental noise, all the measurements were performed within the semi-anechoic chamber of the A3lab group (Dept. of Information Engineering, UNIVPM) as visible in Figure 2.

Several experiments were performed, considering the driver and passenger positions. The measurements performed with OPS (with input samples having Gaussian and uniform distribution) and PPSs for LN and WN filters were compared with measurements based on MLSs and exponential sweeps.

The OPSs have memory length N=2048 samples, order K=3, diagonal number D=3, period P=262,144. The OPS input samples are also quantized in the set [−512:+512]/512. The PPSs for WN and LN filters have N=2048, K=3, D=3, and P=262,120 (to have a period comparable with the OPS) and the samples are represented 24 bits. The MLSs have period $2^{18}-1$. The exponential sweeps have length 262,144 and sweep from around 20 Hz till 22,050 Hz. The sampling frequency is 44,100 Hz. The same power has been considered for all input signals. Additionally, the power consumption and the computational complexity for measuring the HRTF are practically the same for all methods.

**Figure 3:**
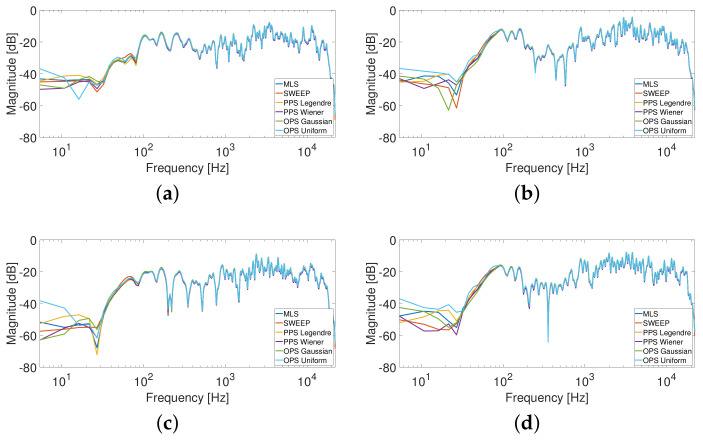
HRTFs measurement at driver position, where (**a**) is the response of the left ear from the left speakers, (**b**) is the response of the left ear from the right speakers, (**c**) is the response of the right ear from the left speakers, and (**d**) is the response of the right ear from the right speakers. A smoothing of 1/12 octave band was applied to the responses.

**Figure 4:**
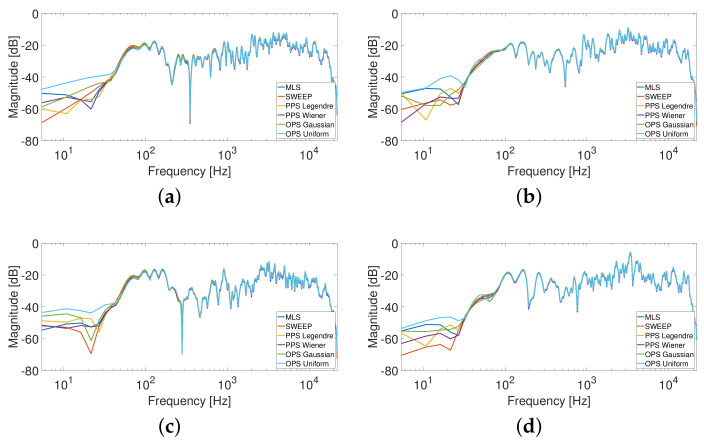
HRTF measurements with the mannequin in the passenger position; in particular, (**a**) is the response of the left ear and the left speakers, (**b**) is the response of the left ear and the right speakers, (**c**) is the response of the right ear and the left speakers and (**d**) is the response of the right ear and the right speakers. A smoothing of 1/12 octave band was applied to the responses.

Figure 3 shows the results obtained for the driver position while Figure 4 shows the results for the passenger position in terms of MLS, Sweep, PPS and OPS. All methodologies present similar results due to the quasi-linear characteristics of the car audio system and of the car environment. These results are in line with the results obtained in [39] for the specific application of room response identification. For this reason, we decided to force a more non-linear behavior introducing external nonlinear devices in the acquisition chain. These results will be reported in the next section.

### 3.2. Emulated Scenario

**Figure 5:**
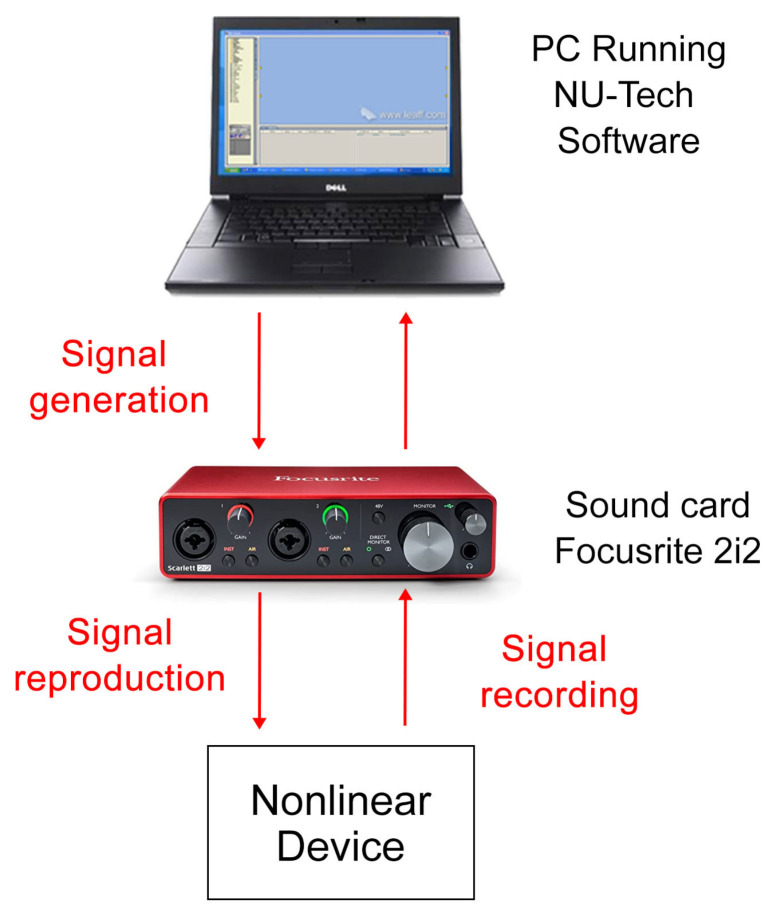
Block diagram of the acquisition procedure for the emulated scenario.

In order to evaluate the performances of the proposed approach with different levels of nonlinearity and to study the effect of noise, two different emulated systems have been considered. Figure 5 shows the procedure adopted for creating the nonlinear signals. In particular, the input signals (i.e., OPS, PPSs, MLSs, and Sweeps signals) of the previous experiment were applied to a nonlinear device exploiting Focusrite Scarlett 2i2 audio interface. Then, the recorded output was convolved in the PC with four HRTFs of 8192 samples previously measured inside the real car environment. Specifically, the HRTFs were those measured in the driver position with the exponential sweep in the previous experiment.

In what follows, the different methods will be compared in terms of log-spectral distance (LSD) between the measured impulse response and the impulse response at the lowest distortion setting with an exponential sweep having 524,288 samples and sweeping from around 20 Hz till 22,050 Hz. The LSD is defined in a band $B=[k_{1}\frac{F_{S}}{T},k_{2}\frac{F_{S}}{T}]$, with $k_{1}$ and $k_{2}\in N$, $F_{S}$ the sampling frequency and *T* the number of samples of the discrete Fourier transform (DFT), as follows:

(11) $$LSD=\sqrt{\frac{1}{k_{2}-k_{1}+1}\sum_{k=k_{1}}^{k_{2}}{\left[10\log_{10}\frac{{\left|H\left(k\right)\right|}^{2}}{|\hat{H}{\left(k\right)|}^{2}}\right]}^{2}},$$

where H(k) is the reference HRFT and $\hat{H}\left(k\right)$ is the measured HRTF. In the experiment, the LSD was measured in the band [100,18000] strictly inside the pass-band.

#### 3.2.1. First Experiment

**Figure 6:**
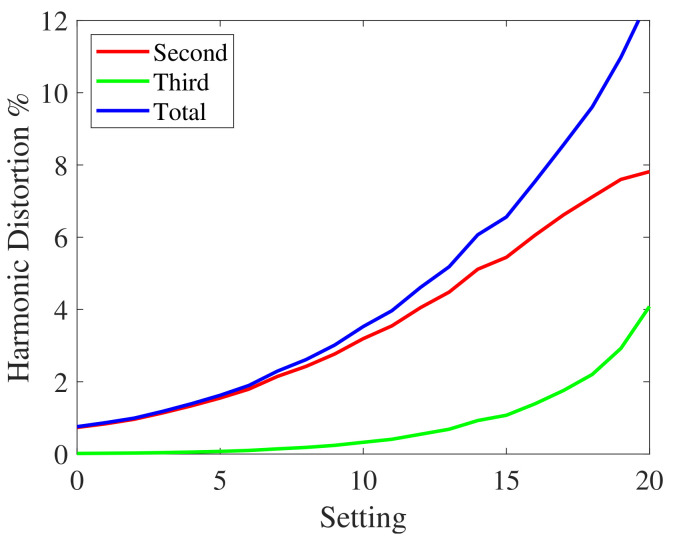
Second, third, and total harmonic distortion of the MIC-100 preamplifier at the different settings.

In this first experiment, the nonlinear device of Figure 5 was a Behringer MIC 100 vacuum tube preamplifier [1]. In the preamplifier, a potentiometer was used to select different levels of nonlinear distortion. In particular, twenty-one different settings were evaluated. Each setting corresponds to a different distortion level. The nonlinearities in the measurement system can be detected and characterized by measuring the harmonic distortion. The second, the third, and the total harmonic distortion on a tone at 1 kHz at the different settings are shown in Figure 6. The curves were obtained by applying input signals with the same power. The harmonic distortion represents the percentage ratio between the power of a harmonic (or all harmonics in case of total distortion) and one of the fundamental frequencies. In order to stress the robustness towards nonlinearities of the different methods, harmonic distortions greater than those normally found in an impulse response measurement system have been considered. In Figure 6, the second-order nonlinearities exhibit higher harmonic distortion.

**Figure 7:**
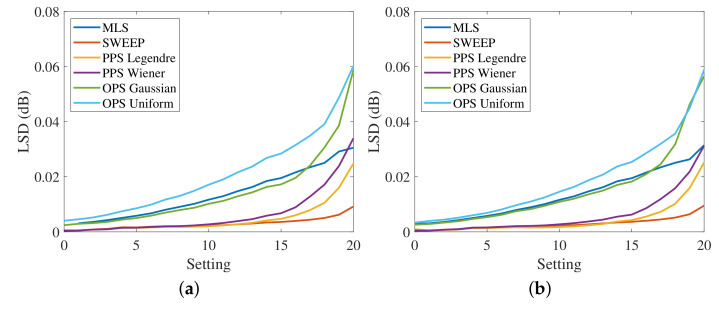

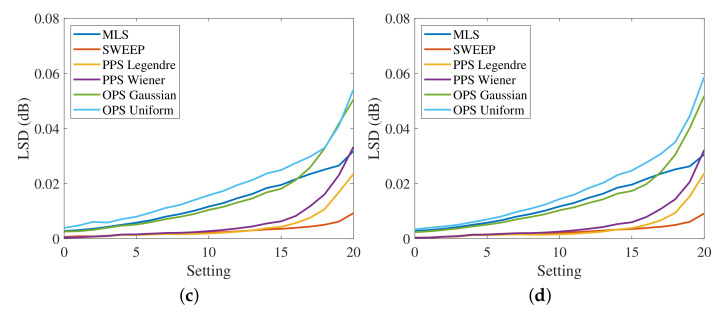
Log-spectral distance in the band [100, 18000] Hz at the different settings with the MIC-100 preamplifier for (**a**) the response of the left ear from the left speakers, (**b**) the response of the left ear from the right speakers, (**c**) the response of the right ear from the left speakers and (**d**) the response of the right ear from the right speakers, and no artificially added noise, i.e., with SNR larger than 60 dB.

**Figure 8:**
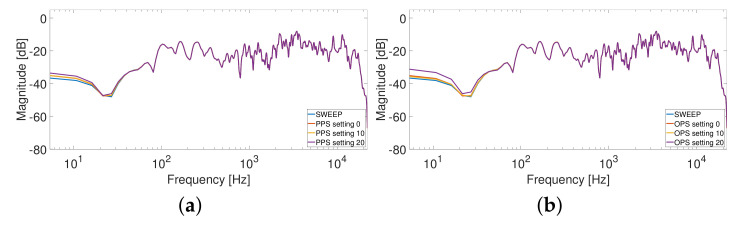
HRTFs measurement at driver position from the left loudspeaker to the left ear, comparing different distortion settings (0,10, and 20) using the MIC-100. The sweep is compared to (**a**) PPS Legendre and (**b**) OPS Gaussian. A smoothing of 1/12 octave band was applied to the responses.

Figure 7 shows the measured LSD for the four HRTFs, varying the settings, without artificial noise added to the output. The SNR is greater than 60 dB, since the noise generated by the power preamplifier is the only existing noise of the system. In this case, the LSD values of the different methods are very similar at the lowest settings, where the distortion is reduced, while they are more different for larger distortions. The desired trend of the LSD varying the settings is a flat curve because it means that the method is immune to nonlinearities. Therefore, the best results are obtained with the exponential sweep that guarantees almost the same value of the LSD for all the settings. The PPSs also show good results, similar to those of the exponential sweep, except for high distortions where the LSD is slightly higher. This aspect is clearly visible in Figure 8a, which shows the measured HRTFs with PPSs compared with the sweep result for settings (0,10, and 20). The OPSs and MLSs exhibit the worst results since the LSD presents a large increase for high levels of distortion. However, the LSD values are small and the difference in the resulting impulse responses is hardly visible. Figure 8b shows the measured HRTFs on the driver position in terms of sweep and OPS.

**Figure 9:**
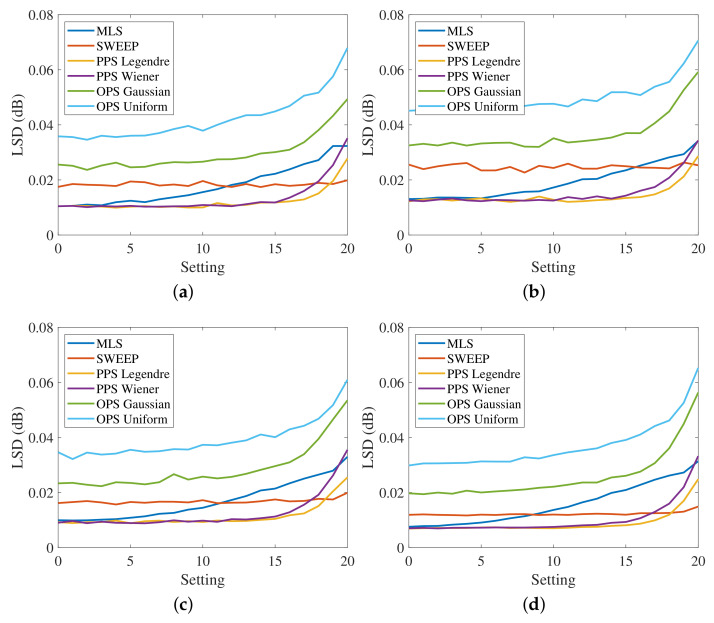
Log-spectral distance in the band [100, 18000] Hz at the different settings with the MIC-100 preamplifier for (**a**) the response of the left ear from the left speakers, (**b**) the response of the left ear from the right speakers, (**c**) the response of the right ear from the left speakers, and (**d**) the response of the right ear from the right speakers with a 40 dB output noise.

Subsequently, white Gaussian noise was added to output signals, resulting in an SNR of 40 dB, in order to evaluate the effect of noise. Each measurement was repeated 100 times using a different output noise every time and the final LSDs values were obtained by the average of the 100 repetitions. Figure 9 shows the obtained results. In this case, the curves related to the OPSs are higher because of a noise gain bigger than 1. In fact, the noise gain of OPS with Gaussian input is 8.8 dB, while the noise gain of OPS with uniform input is 12.5 dB. These values are so large because of the short length of the OPS sequences. The length is similar to the one used for HRTF measurements, but the effect of noise on short-length OPSs is more evident than on the longer-length OPSs used for room impulse response (RIR) measurements [40]. Looking at Figure 9, the best results are presented by the PPSs that have a noise gain of 1, while the exponential sweep is more sensitive to noise, as proved by the raised curve of the LSD.

#### 3.2.2. Second Experiment

**Figure 10:**
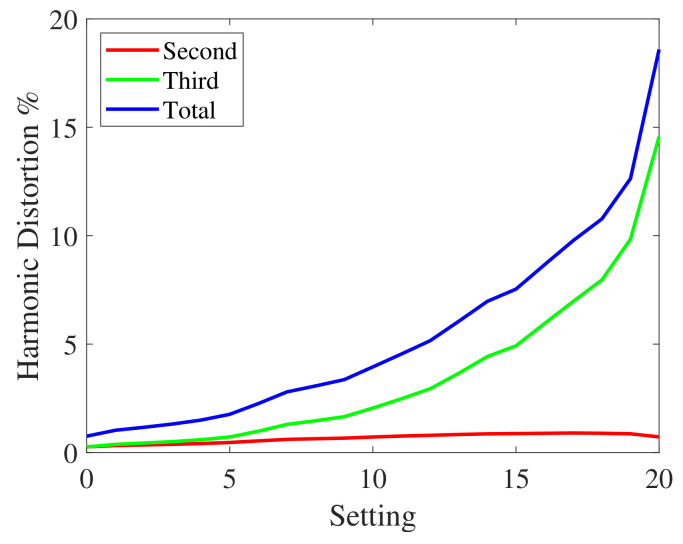
Second, third, and total harmonic distortions of the Electro Harmonix East River Drive pedal at the different settings.

In this second experiment, the nonlinear device of Figure 5 was a guitar pedal Electro Harmonix East River Drive. The guitar pedal had three knobs, one for the volume (set at 50%), one for the tone (set at 100%), and one that allows setting different levels of nonlinear distortion. Twenty-one different settings were considered, and Figure 10 shows the second, third, and total harmonic distortions calculated as in the previous section. Also in this case, many of the harmonic distortions of Figure 10 are much greater than those normally found in an impulse response measurement system, but, as underlined before, the objective of this experiment was to stress the robustness towards the nonlinearities of the different methods. Note that in this experiment the third-order nonlinearities prevail, differently from the previous experiment.

**Figure 11:**
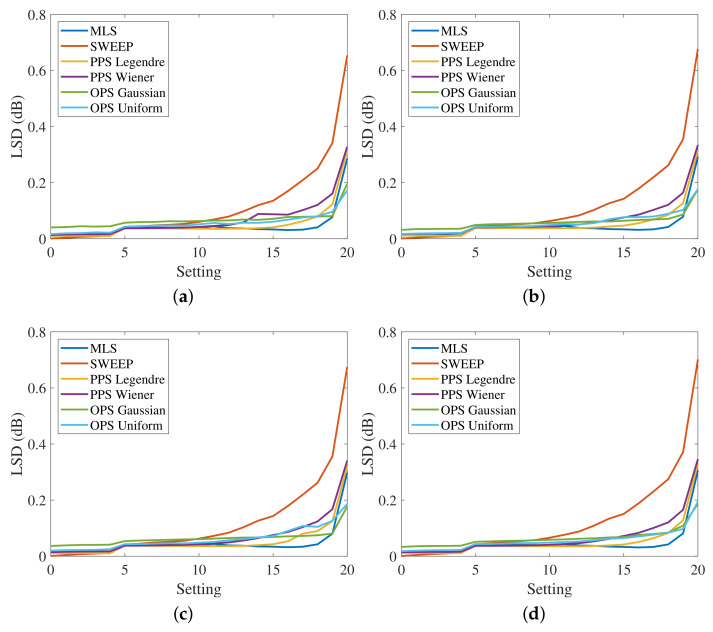
Log-spectral distance in the band [100, 18000] Hz at the different settings with the Electro Harmonix East River Drive pedal for (**a**) the response of the left ear from the left speakers, (**b**) the response of the left ear from the right speakers, (**c**) the response of the right ear from the left speakers and (**d**) the response of the right ear from the right speakers, and no artificially added noise, i.e., with SNR around 60 dB.

**Figure 12:**
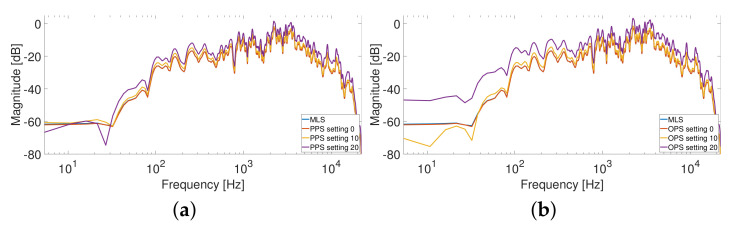
HRTFs measurement at driver position from the left loudspeaker to the left ear, comparing different distortion settings (0,10, and 20) using the Electro Harmonix East River Drive pedal. The MLS is compared to (**a**) PPS Legendre and (**b**) OPS Gaussian. A smoothing of 1/12 octave band was applied to the responses.

Figure 11 shows the LSD measured at the different settings for the four HRTFs when no artificial noise is added to the output. Also in this case, the only noise in the system is that generated by the guitar pedal and the SNR is around 60 dB. In these conditions, except for the exponential sweep signal, all the curves are very close to each other, especially for low distortions at the lowest settings. As pointed out before, the more flat the curve, the more immune is the method to the nonlinearities. Considering this aspect, the worst results are provided by the exponential sweep because this signal is very sensitive to the third-order nonlinearities as reported in the state-of-the-art [25]. The best results are provided by the MLSs’ signal, which originates an almost horizontal curve for most of the settings. The PPSs provide results very similar to the MLSs and only show slightly worse results for very high distortions. In these conditions, the OPSs also show results similar to those of MLSs and PPSs with an increasing LSD for increasing distortion. To underline these aspects, Figure 12 shows the comparison between the measured HRTFs on the driver position in terms of MLS, PPS, and OPS, when the maximum distortion (setting 20) is applied by the guitar pedal Electro Harmonix East River Drive.

**Figure 13:**
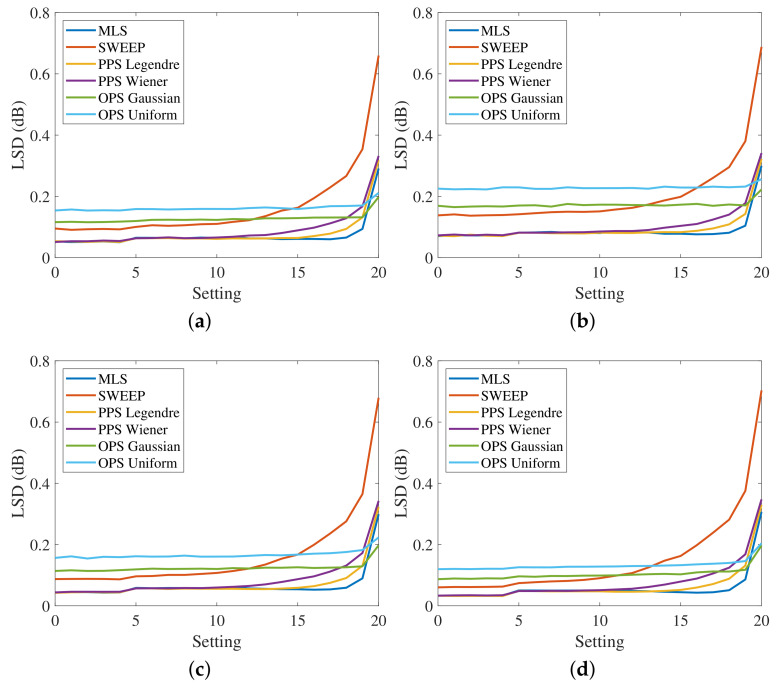
Log-spectral distance in the band [100, 18000] Hz at the different settings with the Electro Harmonix East River Drive pedal for (**a**) the response of the left ear from the left speakers, (**b**) the response of the left ear from the right speakers, (**c**) the response of the right ear from the left speakers, and (**d**) the response of the right ear from the right speakers with a 40 dB output noise.

To study the effect of noise, a white Gaussian noise was added to the output signals used in the measurement to reach a 40 dB SNR. Each measurement was repeated 100 times with a different output noise and the resulting LSDs values were averaged. Figure 13 shows the results obtained in these conditions. The rise of the curves obtained with the OPSs is immediately evident, thus confirming the results obtained in the previous section. Additionally, the exponential sweep measurements result in being more sensitive to noise as can be appreciated from the curve’s rise at all settings, confirming the observations of the previous section. The best results are obtained by the MLS and PPS signals that are able to keep low values at almost all configurations.

## 4. Conclusions

In this paper, an extended evaluation of the novel HRTFs measurement method robust towards nonlinearities proposed in [1] has been presented, by adding new measurements and testing the system with other types of nonlinearities. Using perfect or orthogonal periodic sequences as input, the HRTFs were calculated by the cross-correlation between the output signal and the relative input sequence. In the experiments, two scenarios were taken into account. Firstly, a real scenario was considered and the proposed approach was tested using a car equipped with a binaural mannequin. In this case, small nonlinearities have demonstrated the validity of the approach in comparison with other state-of-the-art methods. In the second scenario, two emulated systems were considered to evaluate the performance of the proposed method, varying the level of nonlinear distortion and the added output noise. Experimental results have proven the robustness of PPSs and OPSs towards nonlinearities. However, short-length OPSs are more sensitive to output noise, showing an increase in the LSD values when noise is added to the output. For this reason, PPSs represent the best solution for the development of an HRTFs measurement method that is robust towards both noise and nonlinearities.

## Figures and Tables

**Table 1:** Summary of HRTFs measurement methods proposed in the literature by category and their robustness towards nonlinearities.

Methods Based on	Robustness towards Nonlinearities	References
Pseudo-random sequences	No	[7,8,9,10,11,12,13,14,15,16]
Adaptive filtering	No	[27,28,29]
Sweep signals	Yes but memoryless	[17,18,19,20,21]
PPSs	Yes with memory	[33,35,36,38]
OPSs	Yes with memory	[39,40]

## Data Availability

PPSs can be downloaded from https://www2.units.it/ipl/res_OPSeqs.htm and OPSs from https://www2.units.it/ipl/res_OPSeqs.htm.
